# Ultrathin MEMS thermoelectric generator with Bi_2_Te_3_/(Pt, Au) multilayers and Sb_2_Te_3_ legs

**DOI:** 10.1186/s40580-020-0218-x

**Published:** 2020-03-03

**Authors:** Yang Liu, Erzhen Mu, Zhenhua Wu, Zhanxun Che, Fangyuan Sun, Xuecheng Fu, Fengdan Wang, Xinwei Wang, Zhiyu Hu

**Affiliations:** 1 National Key Laboratory of Science and Technology on Micro-Nano Fabrication, Shanghai Jiao Tong University, Shanghai, 200240 China; 2 Institute of Nano-Micro Energy, Shanghai Jiao Tong University, Shanghai, 200240 China; 3 Department of Micro-Nano Electronics, Shanghai Jiao Tong University, Shanghai, 200240 China; 4 Institute of Engineering Thermophysics, Chinese Academy of Sciences, Beijing, 100190 China; 5 Center for Advanced Electronic Materials and Devices (AEMD) of Shanghai Jiao Tong University, Shanghai, 200240 China; 6 College of Pipeline and Civil Engineering, China University of Petroleum (East China), Qingdao, 266580 China

**Keywords:** MEMS, Multilayers, TE devices, Bi_2_Te_3_

## Abstract

Multilayer structure is one of the research focuses of thermoelectric (TE) material in recent years. In this work, n-type 800 nm Bi_2_Te_3_/(Pt, Au) multilayers are designed with p-type Sb_2_Te_3_ legs to fabricate ultrathin microelectromechanical systems (MEMS) TE devices. The power factor of the annealed Bi_2_Te_3_/Pt multilayer reaches 46.5 μW cm^−1^ K^−2^ at 303 K, which corresponds to more than a 350% enhancement when compared to pristine Bi_2_Te_3_. The annealed Bi_2_Te_3_/Au multilayers have a lower power factor than pristine Bi_2_Te_3_. The power of the device with Sb_2_Te_3_ and Bi_2_Te_3_/Pt multilayers measures 20.9 nW at 463 K and the calculated maximum output power reaches 10.5 nW, which is 39.5% higher than the device based on Sb_2_Te_3_ and Bi_2_Te_3_, and 96.7% higher than the Sb_2_Te_3_ and Bi_2_Te_3_/Au multilayers one. This work can provide an opportunity to improve TE properties by using multilayer structures and novel ultrathin MEMS TE devices in a wide variety of applications.

## Introduction

Fossil fuels shortages and pollution of environment have raised the attention of researches in recent years. The diversification and efficient multi-level utilization of the energy become important technical approaches to solve these energetic and environmental problems. Thermoelectric (TE) devices show an inherent superiority harvesting the energy generated from waste heat and low quality thermal energy and they constitute a promising way to supply power [1–3]. In addition, TE conversion has particular advantages such as small size, good output quality, no running noise, no pollution, and wide operating temperature range [4–6]. Consequently, TE devices are widely used in wearable devices, vehicles, industrial waste-heat recovery systems and solar energy systems to convert waste heat into electrical energy [7–9]. However, the current conversion efficiency of TE devices is much lower than that of other mechanical systems. The high-efficiency and multi-stage utilization of industrial waste heat, environmental energy recovery, and the development of special power supplies are an urgently needed for the development of TE technologies. Improving the conversion efficiency of TE equipment is a significant challenge in this field. Increasing the conversion efficiency of TE devices corresponds to the enhancement of the performance of the TE materials [10, 11]. The properties of such materials can be determined via the dimensionless figure of merit *ZT*, which is defined as *S*^*2*^·*σ*·*T*·*κ*^*−1*^[12], where *S, σ, T, and κ* correspond to the Seebeck coefficient, the electrical conductivity, the absolute temperature and the thermal conductivity, respectively. Alternatively, the performance can be also evaluated by using the power factor, *PF* = *S*^*2*^·*σ*. In order to boost the thermoelectric conversion efficiency, various approaches to enhance the *ZT* value have been proposed and developed.

In the 1990s, Hicks and Dresselhaus showed that low-dimensional TE materials, such as quantum wires and quantum wells, exhibit a significantly high *ZT* values [13, 14]. Using low-dimensional nanostructures to selectively alter the properties of TE materials has been proven to be a new technique to enhance their thermoelectric properties by customizing both their electron and phonon transmission and their scattering characteristics [15–17]. Sun et al. improved the thermoelectric properties of n-type Bi_2_Te_2.7_Se_0.3_ thin films via the introduction of Pt nanoinclusions triggered by pulsed laser deposition [18]. Sumithra et al. introduced semimetal nanoinclusions into Bi_2_Te_3_ samples and achieved an enhanced ZT [19].

Although scientists have obtained TE materials with high *ZT* and *PF* by using low-dimension and nanostructures, only the performance of most of these materials has been tested but they have not been yet used for practical applications. With the development of Microelectromechanical Systems (MEMS), the preparation and the application of various micro-devices have gradually become a reality. It is more convenient to fabricate low-dimensional TE materials to develop MEMS thermoelectric devices. The micro-nano processing significantly improves the amount of TE modules in a single device, increasing its power output. Therefore, miniaturization is a useful way to enhance the performance of TE devices and to achieve superior quality of integration. Jeffrey et al. fabricated a thermoelectric microdevice containing 126 TE modules via a MEMS-like electrochemical process [20]. A TE device consisting of more than 46,000 (Bi, Sb)_2_Te_3_ TE modules electronically connected in series was developed [21]. Highly integrated, ultra thin thermoelectric devices can generate electricity upon a minimal temperature difference, and can convert low-quality thermal energy into electricity.

In this paper, 800 nm n-type Bi_2_Te_3_, Bi_2_Te_3_/Pt, Bi_2_Te_3_/Au multilayers and p-type Sb_2_Te_3_ films were prepared and their corresponding TE properties were evaluated. Moreover, three ultrathin TE devices were fabricated by combining different types of the films. To the authors’ knowledge, this is the first time that < 1 μm-TE modules have been build. TE conversion properties of the Sb_2_Te_3_, Bi_2_Te_3_ and Bi_2_Te_3_/(Au, Pt) multilayer thin films were evaluated. In addition, the TE conversion properties were investigated via experiments and theoretical analyses.

## Experiments and methods

### Fabrication of the TE thin films and MEMS thermoelectric devices

The TE thin films were prepared by alternate sputtering in a high-vacuum magnetron sputtering system at room temperature. The TE multilayers are composed of alternating layers of Bi_2_Te_3_ and metal (Pt, Au) with a thickness of 20 and 5 nm with 40 periods, respectively. The sputtering mode used was the radio frequency (RF) method for Sb_2_Te_3_ and Bi_2_Te_3_ and direct current (DC) technique for Pt and Au. To ensure deposition uniformity, the substrate was rotated at a speed of 20 rpm.

The MEMS thermoelectric devices were fabricated on a monocrystalline silicon wafer covered by a SiO_2_ layer via a lithography process. Sb_2_Te_3_ as the p-type TE modules and Bi_2_Te_3_, Bi_2_Te_3_/Pt, and Bi_2_Te_3_/Au as the n-type TE modules were selected to fabricate the samples. A sheet of 20 nm Cr was deposited as a bonding layer during the fabrication process. Copper was selected as the electrode material due to its thermal conductivity, outstanding electro-conductivity, and its wide use in the currently developed TE devices. Five photomasks containing a graphical architecture were used to prepare the MEMS devices with 572 TE modules in series. Additional file 1: Fig. S1 shows schematically the basic steps of the fabrication process of the MEMS devices. The details of the fabrication method are initially proposed and then, demonstrated as a hybrid fabrication technique for ultrathin thermoelectric devices in a previous work of this research group [22]. In order to prevent the oxidation of copper in the electrode, the sacrificial photoresist was not removed in these MEMS devices. Moreover, this material serves as a supporting structure to ensure that the top electrodes are well interconnected.

### Characterizations

The Seebeck coefficient of the Sb_2_Te_3_, Bi_2_Te_3_ and Bi_2_Te_3_/(Au, Pt) multilayers was characterized by using a home-built system. The details of this apparatus are described in a previous study [23]. Furthermore, an oven was used to provide the test ambient temperature. A temperature difference (ΔT) was applied to the both ends of TE films via two Peltier plates, which were controlled by using an adjustable regulated DC power supply. The value of ΔT was varied in the 1–4 K range. The voltage difference (ΔV) was measured by using a data acquisition setup. The Seebeck coefficient was obtained by using the relation S = ΔV·ΔT^−1^. The representative ΔV − ΔT curves, which were used for the extraction of the Seebeck coefficient are shown in Additional file 1: Fig. S2. The surface morphology and the cross-section microstructures of the fabricated MEMS devices and multilayer thin films were characterized via field emission scanning electron microscopy (SEM). Moreover, both the unannealed and annealed thermoelectric Bi_2_Te_3_ and Bi_2_Te_3_/(Au, Pt) multilayer thin films were analyzed via Poly-functional X-Ray Diffractometry (XRD) and by using the Hall Effect Measurement System (model: MMR). The thermal conductivity of the annealed TE thin films was obtained via the time-domain thermoreflectance (TDTR) method [24]. The electric power generated by the MEMS devices was measured using a heater and a data acquisition system. The schematic diagram of such devices is shown in Additional file 1: Fig. S3. In order to avoid the oxidation of the copper electrodes, TE devices were only heated up to 463 K.

## Results and discussion

### Characterizations of the TE thin films

**Fig. 1:**
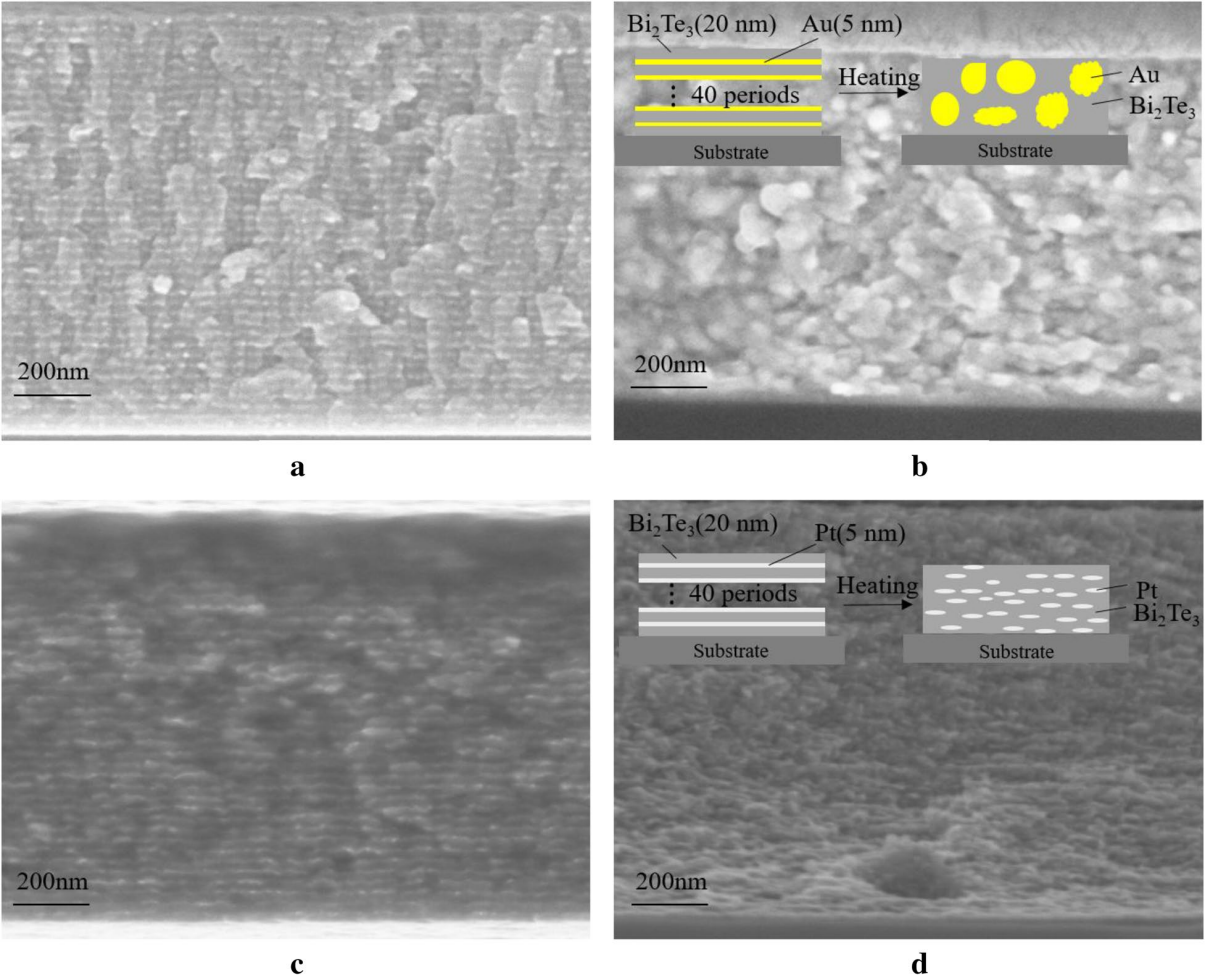
Cross-section SEM images of the multilayer thin films **a** Bi_2_Te_3_/Au, **b** annealed Bi_2_Te_3_/Au, **c** Bi_2_Te_3_/Pt, **d** Annealed Bi_2_Te_3_/Pt. The insets in **b** and **d** show the schematic transformation of the Bi_2_Te_3_/Au and Bi_2_Te_3_/Pt multilayers as a function of the temperature, respectively

The SEM images of the cross-section of the multilayer thin films before and after the annealing process are shown in Fig. 1. The films were annealed at 473 K for 2 h to simulate the TE modules in the MEMS devices and their photoresist pre-bake process and hard baking, which are involved in their preparation. Figure 1a and c show a fuzzy delamination of both the metal and Bi_2_Te_3_ compounds in non-annealed multilayers, respectively. However, the multilayer structure disappears upon annealing and this inevitably influences the properties of the materials. It can be seen from the insets in Fig. 1b and d that Au agglomerates to form large particles after annealing, whereas Pt tends to be uniformly distributed in the Bi_2_Te_3_. Moreover, intermittent lamellar structures are still present inside the Bi_2_Te_3_/Pt multilayers. This phenomenon can be attributed to the different thermal expansion coefficients of Au and Pt.

The effects of the pressure on the material can be neglected in a solid, and the volumetric thermal expansion coefficient (*α*_*v*_) can be written as follows:

1 $$\alpha_{v} = \frac{1}{v}\frac{\Delta v}{{\Delta T}}$$

where *v* is the volume of the material, *Δv* corresponds to the volume difference, and *ΔT* is the temperature difference. At 293 K, the volumetric coefficients of Au and Pt measure 2.7 × 10^−5^ K^−1^ and 4.2 × 10^−5^ K^−1^ respectively [25]. The initial volume of the two multilayer structures is identical, then the volume of the Bi_2_Te_3_/Au multilayers becomes about 1.6 times larger than that of the Bi_2_Te_3_/Pt multilayers during annealing. This leads to the destruction of the multilayer structure in the Bi_2_Te_3_/Au samples. Moreover, Au agglomerates more easily when the multilayer structure is broken. For this reason, many large particles appear in the annealed Bi_2_Te_3_/Au multilayers, resulting in a decrease of the compound performance.

**Fig. 2:**
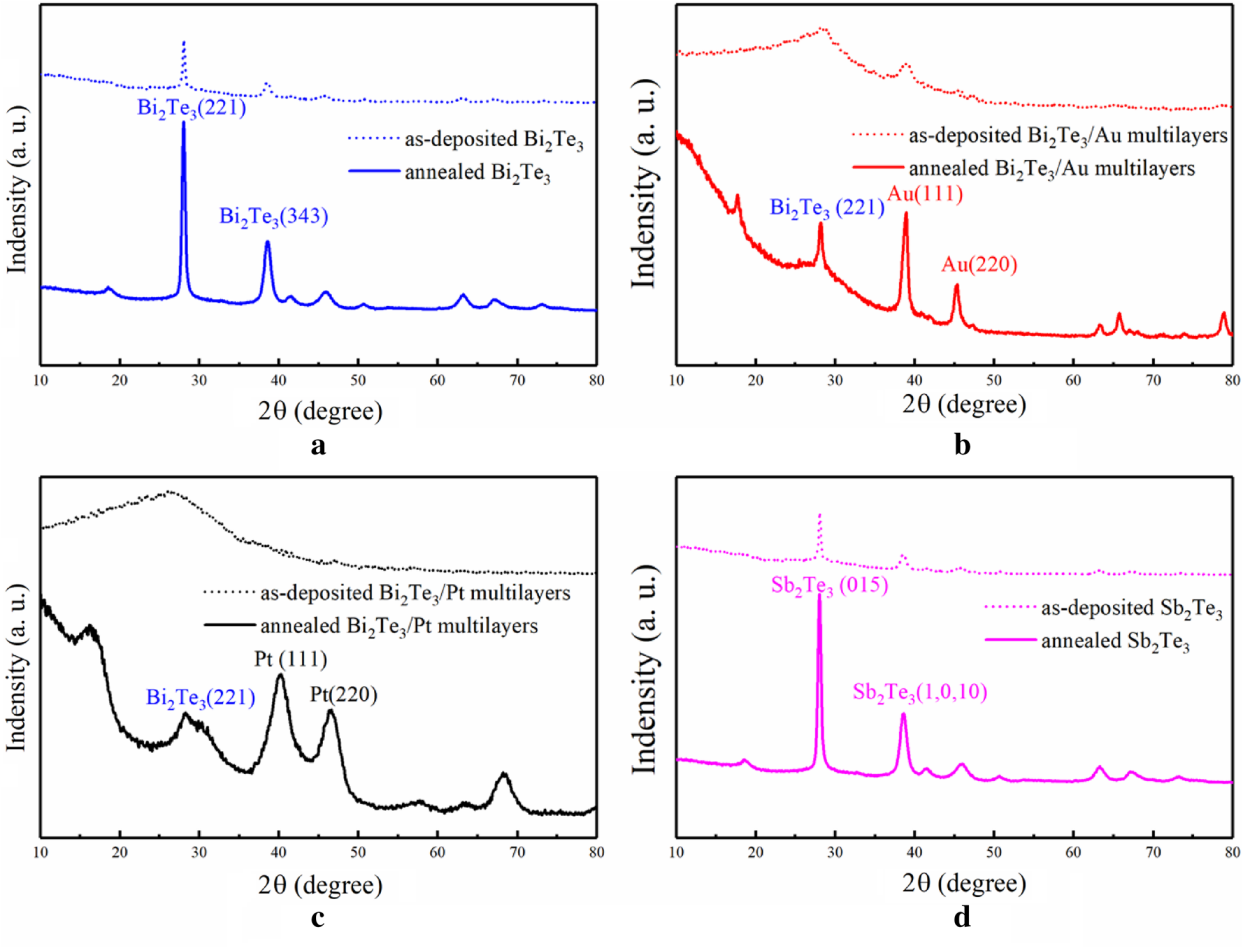
XRD patterns of the films, **a** Bi_2_Te_3_/Au, **b** Bi_2_Te_3_/Au, **c** Bi_2_Te_3_/Pt, **d** Sb_2_Te_3_

The XRD patterns of the as-deposited and annealed films are presented in Fig. 2. No characteristic peaks derived from additional materials can be observed. Only the Au, Pt, and Bi_2_Te_3_ features can be detected in the XRD patterns of the annealed multilayers, indicating that the materials form interstitial solid solutions rather than compounds during the annealing process. In the XRD patterns of the non-annealed multilayers, the characteristic peak of Bi_2_Te_3_ with low crystallinity is present, whereas the other two multilayers are amorphous. After annealing under the identical conditions, the pure Bi_2_Te_3_ compound exhibits a higher crystallinity than the multilayers material. This implies that the compound was difficult to crystallize due to the presence of the metal particles.

Estimating the average crystallite size by using the Debye–Scherrer formula [26]:

2 $$D = \frac{K \cdot \lambda }{{B\cos \theta }}$$

where *D* is the average grain size, *K* is a dimensionless factor with a value of 0.89, *λ* is the X-ray wavelength, *B* corresponds to the line broadening at half of the maximum intensity, and *θ* is the Bragg angle. An average grain size of 9.1 nm, 16.2 nm, and 4.0 nm was obtained for the Bi_2_Te_3_, Bi_2_Te_3_/Au, and Bi_2_Te_3_/Pt multilayers materials, respectively. These results are in agreement with the SEM images.

**Fig. 3:**
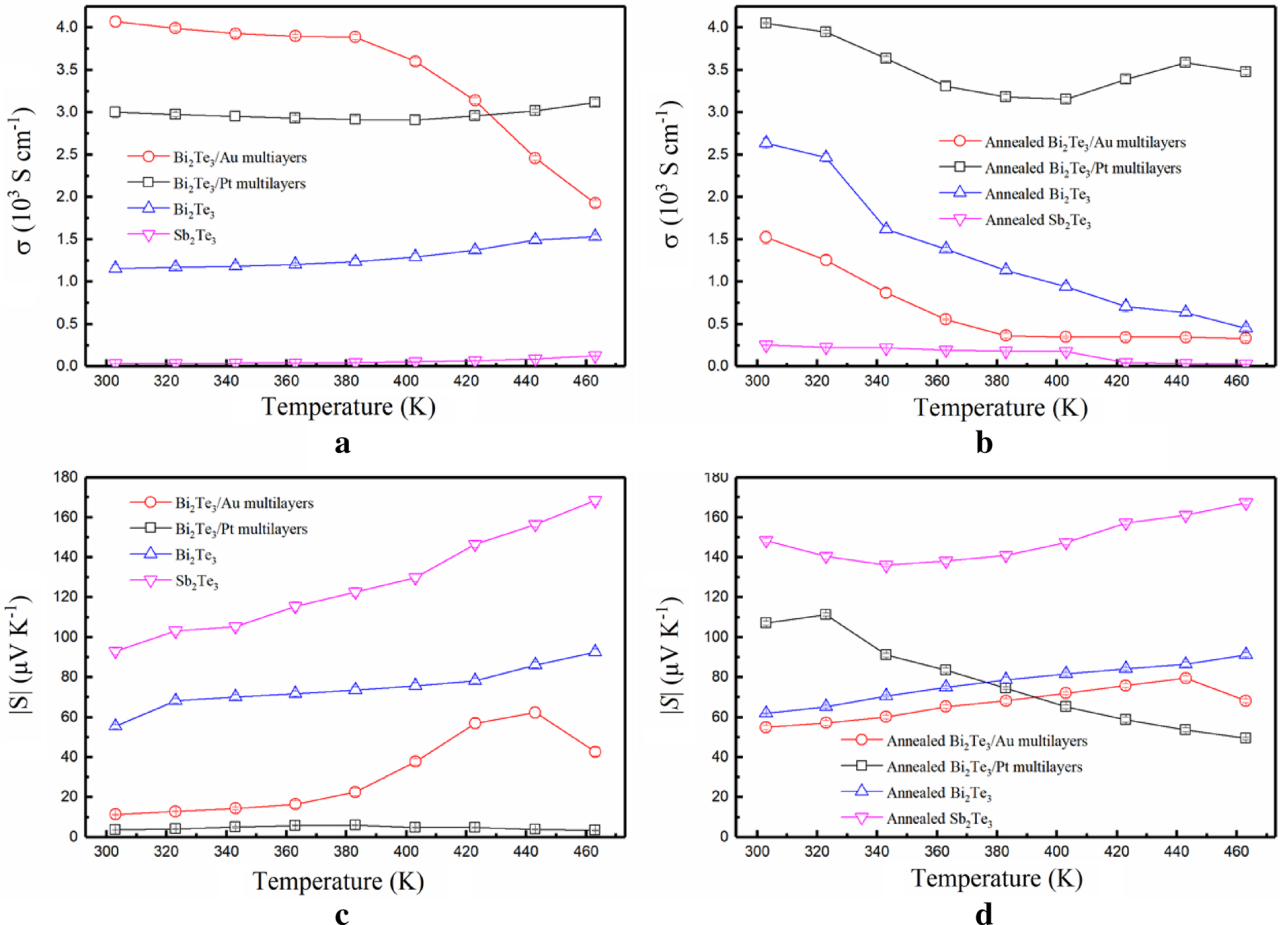
Temperature dependence of the in-plane **a** electrical conductivity, *σ*, of the deposited films; **b***σ* of the annealed films; **c** absolute Seebeck coefficient, |*S*|, of the deposited films; **d** |*S*| of the annealed films

Figure 3 shows the in-plane electrical conductivities and the absolute Seebeck coefficients of the annealed TE film samples in the 303–463 K range. This information helps in understanding their changes upon an increase in the temperature of the multilayer structures. The Sb_2_Te_3_ and Bi_2_Te_3_ samples exhibit normal semiconductor properties, i.e., their conductivity and Seebeck coefficients increase upon an increase in temperature. A metallic-like behaviour is observed in the Bi_2_Te_3_/Au multilayers when Au is introduced. The annealed films show an enhanced conductivity and Seebeck coefficient at 303 K when compared to the non-annealed films. The electrical conductivities of annealed TE films decreases with an increase in temperature while that of Bi_2_Te_3_/Pt multilayers rebounds at 423–443 K, this is because the change of crystalline state is a non-monotony variation.

The carrier mobility and the concentration of the annealed multilayer films (Table. 1) were compared. The results show numerous electrons accumulate in Au close to its energy barrier. This provides the flow of n-type carrier towards the conduction band of Bi_2_Te_3_ when Au starts agglomerating. Moreover, the directional movement of the electrons is limited, leading to a reduction in the electrical conductivity of the Bi_2_Te_3_/Au multilayers upon an increase in temperature. However, due to the higher thermal stability of platinum, the multilayer structure remains almost unchanged in the Bi_2_Te_3_/Pt multilayer film when the temperature is changed. Thus, the Bi_2_Te_3_/Pt multilayers exhibit a stable electrical conductivity temperature profile. The increase in the electrical conductivity of the Bi_2_Te_3_/Pt multilayers is mostly correlated to an increase in mobility.

**Table 1:** Carrier concentration, mobility and TE properties of the annealed films at 303 K

Types of films	Sb_2_Te_3_	Bi_2_Te_3_	Bi_2_Te_3_/Au multilayer	Bi_2_Te_3_/Pt multilayer
Type of carrier	Holes	Electrons	Electrons	Electrons
Carrier concentration [cm^−3^]	(2.9 ± 0.3) × 10^20^	(1.8 ± 0.2) × 10^21^	(6.8 ± 0.1) × 10^21^	(2.0 ± 0.2) × 10^21^
Carrier mobility [cm^2^ V^−1^ s^−1^]	5.7 ± 0.2	8.77 ± 0.4	1.5 ± 0.1	13.2 ± 0.2
Seebeck coefficient [μV K^−1^]	148.4 ± 1.0	62.0 ± 0.3	55.0 ± 1.1	107.05 ± 1.1
Electrical conductivity [S cm^−1^]	252 ± 6	2638 ± 58	1525 ± 81	4054 ± 11
Power factor [μW cm^−1^ K^−2^]	5.5 ± 0.6	10.1 ± 0.4	4.61 ± 0.7	46.5 ± 0.5

In the deposited multilayers, if the metal layer is not broken, the metal layer generates a short cut in the test circuit, leading to a low Seebeck coefficient, as shown in Fig. 3c. The increase of the Seebeck coefficient in the annealed multilayers is generated by the heat of the metal layer, when it is embedded into the semiconductor matrix as a secondary non-inclusion phase. The energy-dependent carrier scattering effect is introduced into the bending band of Fermi level alignment at the metal/semiconductor interface, to scatter low-energy carriers and improve |*S*|, but hardly significantly reducing conductivity [27, 28].

**Fig. 4:**
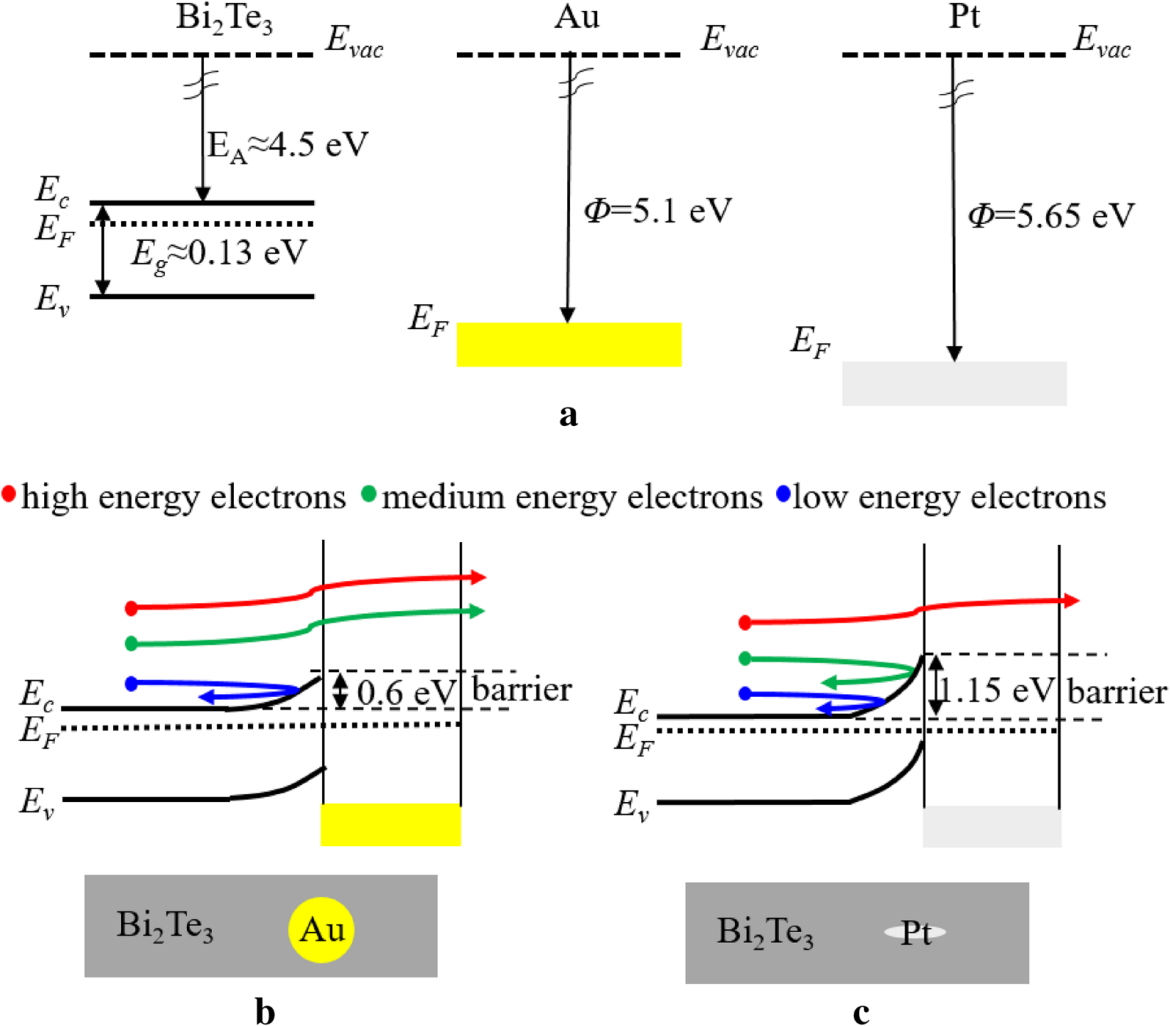
**a** Band energy diagram of Bi_2_Te_3_, Pt, and Au before contact. The band gap, electron affinity, and ionization potential of Bi_2_Te_3_ and the work function of Pt and Au are estimated from their bulk values; **b** Equilibrium band alignment of Bi_2_Te_3_ and Pt after contact; **c** Equilibrium band alignment of Bi_2_Te_3_ and Au after contact. No interface defect states are taken into account

The band alignments of Bi_2_Te_3_, Au and Pt are shown in Fig. 4a. The band gap (*Eg*), the electron affinity (*E*_*A*_) of Bi_2_Te_3_, and the work function (*Φ*) of Au and Pt are estimated from their bulk values [19, 29]. The equilibrium band diagrams of Au-Bi_2_Te_3_ and Pt-Bi_2_Te_3_ at the metal–semiconductor interface are illustrated in Fig. 4b and c, respectively. The results indicate the formation of a band bending potential barrier at the interface. Moreover, no interface defect states (causing the Fermi level pinning) are observed.

As described above, the barrier created by the bending of the Pt/Bi_2_Te_3_ and Au/Bi_2_Te_3_ interfaces promotes a “selective” block of the low energy electrons, causing an increase in the average carrier energy, which leads to an increase of |*S*|. Usually, there is a Schottky barrier at the metal–semiconductor interface, which depands mostly on the difference between the E_A_ of the semiconductor and the work function of the metal. Pt shows to have an advantage due to its higher work function when compared to Au. This generates the formation of a higher Schottky barrier when Pt is combined with the Bi_2_Te_3_ n-type semiconductor (with *E*_*A*_ of 4.5 eV). In this case, the average carrier energy in Bi_2_Te_3_/Pt multilayers is higher and thus, a higher |*S*| is obtained. Moreover, the enhancement of the |*S*| can be expressed via the Mott relation:

3 $$\begin{aligned}{\text{|S|}} &= \frac{{\pi^{2} k_{B}^{2} }}{3q}T\left\{ {\frac{{d\left[ {\ln \left( {\sigma \left( E \right)} \right)} \right]}}{dE}} \right\}_{{E = E_{F} }} \\ &= \frac{{\pi^{2} k_{B}^{2} }}{3q}T\left\{ {\frac{dn\left( E \right)}{{nd_{E} }} + \frac{d\mu \left( E \right)}{{\mu d_{E} }}} \right\}_{{E = E_{F} }}\end{aligned}$$

where *k*_*B*_ is the Boltzmann constant, *T* is the absolute temperature, *q* is the electronic charge, *σ*(*E*) is the electrical conductivity, corresponds to the carrier concentration, and *μ*(E) to the carrier mobility, which depends on the energy. In the Bi_2_Te_3_/Au multilayers, the huge increase in the carrier concentration causes a negative influence, which results in a lower |*S*| value when compared to Bi_2_Te_3_.

The cross-plane thermal conductivity of the annealed Bi_2_Te_3_ is 1.33 W m^−1^ K^−1^, whereas that of the annealed Bi_2_Te_3_/Au and Bi_2_Te_3_/Pt multilayers is 1.39 and 1.22 W m^−1^ K^−1^, respectively. This shows that the disappearance of the multilayer structure in the Bi_2_Te_3_/Au multilayers leads to an increase in their thermal conductivity: the residual multilayer structure of the samples effectively scatters the phonons and reduces the thermal conductivity of the material. The cross-plane thermal conductivity of the annealed Sb_2_Te_3_ sample measures 1.06 W m^−1^ K^−1^.

### Characterizations of the TE devices

**Fig. 5:**
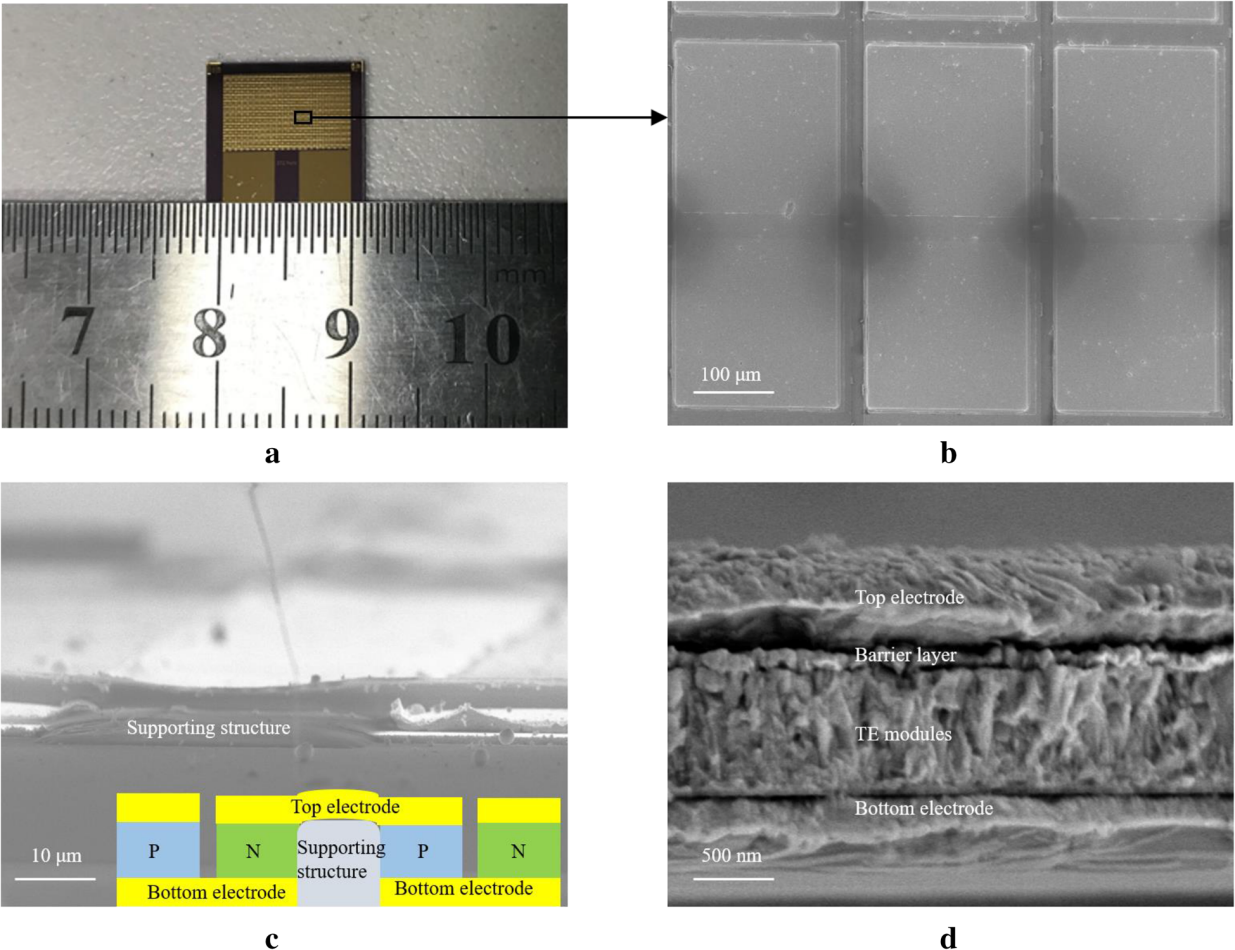
**a** Photograph of an actual fabricated MEMS device; **b** SEM image of the local structure in the MEMS device; **c** SEM cross-sectional view of the cross-plane of the supporting structure; **d** SEM cross-sectional view of the cross plane of a single thermoelectric leg. The inset shown in **c** shows the schematic diagram of the TE devices

To investigate the power-generation capability of the Bi_2_Te_3_/(Au, Pt) multilayers, three types of MEMS thermoelectric devices with the same p-type TE module consisting of Sb_2_Te_3_ and different n-type TE modules were designed and prepared. As an example, here the results of the MEMS devices fabricated with a n-type TE module consisting of pristine Bi_2_Te_3_, Bi_2_Te_3_/Au multilayers, or Bi_2_Te_3_/Pt multilayers (labelled ST-BT, ST-BA, or ST-BP, respectively) are reported. One of the individual devices, consisting of 572 thermoelectric modules, is shown in Fig. 5a. Its SEM images are illustrated in Fig. 5b–d. Figure 5b, c prove that the electric-connected top electrode has been deposited smoothly. Each TE module has a size of 200 × 200 × 0.8 μm, and the dark area in Fig. 5b illustrates the organic support structure. The height of the supporting structure in Fig. 5c is relatively consistent with the thermoelectric column, which ensures the smooth conduction of the top electrode. The inset in Fig. 5c shows the schematic diagram of the TE devices. The barrier layer in Fig. 5d is a layer of gold with a thickness of 100 nm, which exists above the thermoelectric material to prevent the surface of the thermoelectric material from being oxidized or corroded. The small gap between the top electrode and the thermoelectric modules, which one can observe in Fig. 5d, is caused by the stress generated during the sample preparation. The SEM image of the cross plane of a single thermoelectric leg also illustrates that the Bi_2_Te_3_/metal multilayer structure in the MEMS devices disappears and is replaced by a hybrid structure. Hard baking is necessary to build a supporting structure and this results in the diffusion of the metal and Bi_2_Te_3_. This process corresponds to the disappearance of multilayers structure and introduction of metal nano-inclusions. The diffusion in the multilayer structure while heating constitutes a problem, which has to be solved in practical applications of such multilayers. The rough interfaces between the electrode and the TE modules generate a considerable contact thermal resistance, which significantly decreases the temperature difference of the TE module. Thus, by optimizing the structure of devices and TE modules, the performance of TE devices can be significantly enhanced.

**Fig. 6:**
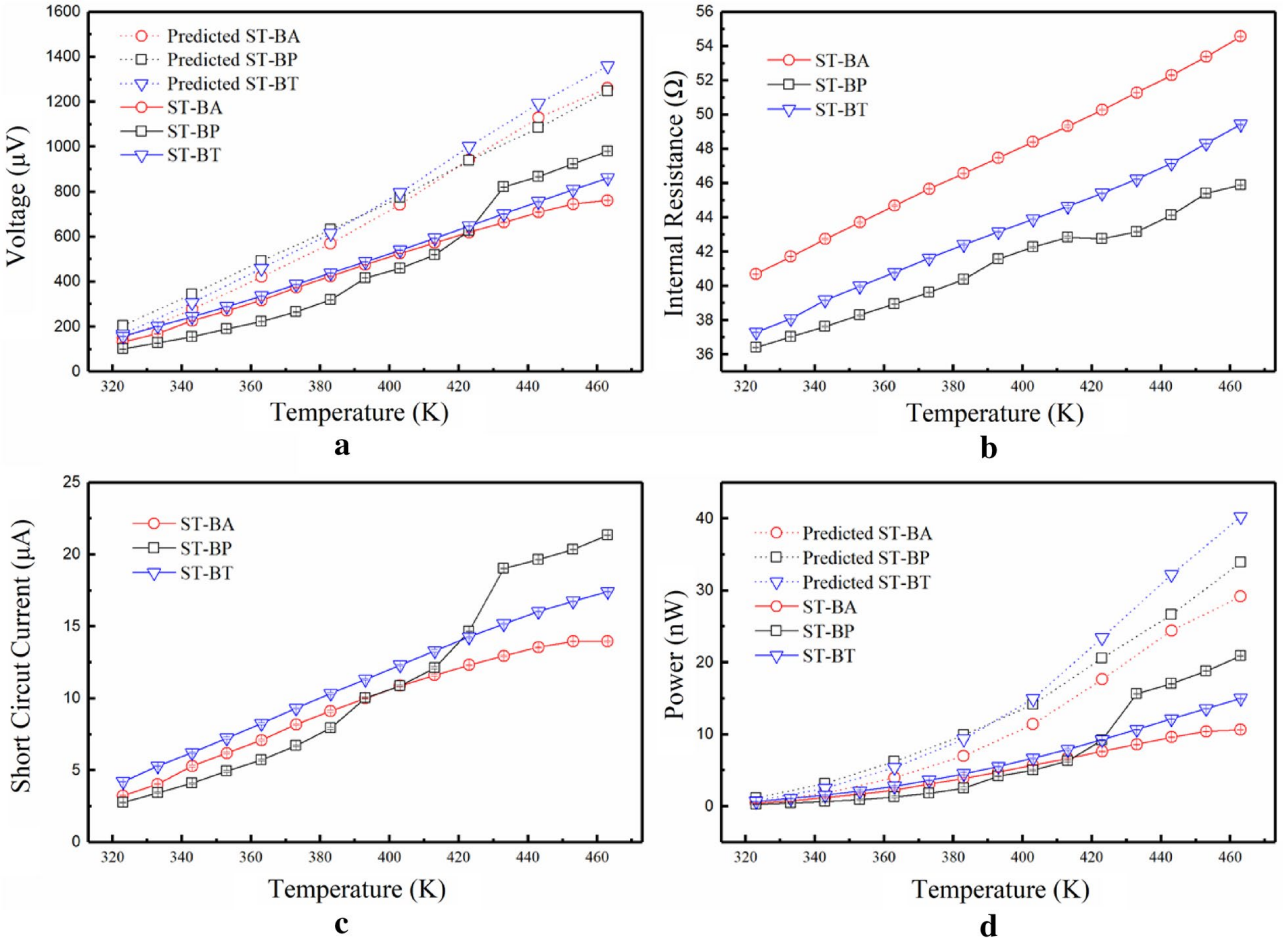
Electrical properties of the TE device measured at various temperatures; **a** Voltage; **b** Resistance; **c** Short circuit current; **d** Output power. The dotted lines in **a** and **d** show the analytical model results

The output characteristics and the internal resistance as a function of the temperature are shown in Fig. 6. From the growth trend of the curve reported in Fig. 6d, it can be deduced that the performance growth of ST-BA tends to stagnate. This may be due to the decrease of the in-plane Seebeck coefficient of the annealed Bi_2_Te_3_/Au multilayers at 463 K and to the isotropic morphology of the annealed Bi_2_Te_3_/Au multilayers. Initially, the performance of ST-BP is lower than that of the other two compounds, but between 413 and 433 K its internal resistance hardly changes during and its short circuit voltage rises sharply. Despite, the experimental limitation in the measurement of the cross-plane |*S*| of thin films, one can speculate that the cross-plane |*S*| of the Bi_2_Te_3_/Pt multilayers increases between 413 and 443 K, due to a Schottky barrier. The power of ST-BP reaches 20.9 nW at 463 K, which corresponds to an enhancement larger than 39.5% when compared to ST-BT. One can conclude that when the external load is equivalent to its internal resistance, the calculated maximum output power of ST-BP reaches 10.5 nW at 463 K, and the output power density is 0.02 W m^−2^ and 2.88 × 10^4^ W m^−3^. In addition, if a forced cold end is added to the existent cold end of the device, the output can be increased by one to two orders of magnitude [22]. The in-plane Seebeck coefficient of the Bi_2_Te_3_/Au multilayers decreases upon an increase of the temperature, leading to the superior properties of the ST-BP material at high temperature. Moreover, these results show that the annealed Bi_2_Te_3_/Pt multilayers are anisotropic.

The TE devices were analyzed by using the method proposed in the Refs [30, 31]. The one-dimensional heat transfer equation applies to all the TE devices and relates the voltage and the temperature difference and the power output to the load. The power output and the voltage of the TE devices can be calculated as follows:

4 $$P = U \cdot I$$

5 $$U = S \cdot \left( {T_{Hs} - T_{Cs} } \right) - Ir$$

where *T*_*Cs*_ and *T*_*Hs*_ corresponds to the temperatures at the cold and hot surfaces, respectively, *I* is the electrical load current, and *r* is the internal electrical resistance. The heat transfer mechanism is based on the Seebeck effect, on the conduction effect, and on the Joule effect in p-type and n-type semiconductor blocks. For this reason, the rate of the heat entering from the hot side of the device, *Q*_*H*_, and the rate of the heat leaving from its cold side, *Q*_*C*_, can be expressed as:

6 $$Q_{H} = S \cdot T_{Hs} \cdot I + \kappa \cdot \left( {T_{Hs} - T_{Cs} } \right) - 0.5I^{2} r$$

7 $$Q_{C} = S \cdot T_{Cs} \cdot I + \kappa \cdot \left( {T_{Hs} - T_{Cs} } \right) + 0.5I^{2} r$$

where *S* is the Seebeck coefficient of the TE legs, *T*_*Hs*_ and *T*_*Cs*_ correspond to the hot and cold surface temperature, and *κ* is the thermal conductivity. If the temperature is inconstant, the energy balance equations between the hot and the cold side of the TE device can be expressed as:

8 $$Q_{H} = h_{1} A_{1} \left( {T_{H} - T_{Hs} } \right) = S \cdot T_{Hs} \cdot I + \kappa \cdot \left( {T_{Hs} - T_{Cs} } \right) - 0.5I^{2} r$$

9 $$Q_{C} = h_{2} A_{2} \left( {T_{Cs} - T_{C} } \right) = S \cdot T_{Cs} \cdot I + \kappa \cdot \left( {T_{Hs} - T_{Cs} } \right) + 0.5I^{2} r$$

where *h*_*1*_ and *h*_*2*_ correspond to the equivalent convection heat transfer coefficients of the hot and cold side, and *A*_*1*_ and *A*_*2*_ are the hot and cold heat transfer surface area of the devices, respectively. By solving Eqs. (6) and (7), *T*_*Hs*_ and *T*_*Cs*_ are expressed as:

10 $$T_{Hs} = \frac{{\left( {\kappa - SI + h_{2} A_{2} } \right)\left( {0.5I^{2} r + h_{1} A_{1} T_{H} } \right) + \kappa \left( {0.5I^{2} r + h_{2} A_{2} T_{C} } \right)}}{{\left( {h_{1} A_{1} + \kappa + SI} \right)\left( {\kappa - SI + h_{2} A_{2} } \right) - \kappa^{2} }}$$

11 $$T_{Cs} = - \frac{{\kappa \left( { - 0.5I^{2} r - h_{1} A_{1} T_{H} } \right) + \left( { - h_{1} A_{1} - \kappa - SI} \right)\left( {0.5I^{2} r + h_{2} A_{2} T_{C} } \right)}}{{\left( {h_{1} A_{1} + \kappa + SI} \right)\left( {\kappa - SI + h_{2} A_{2} } \right) - \kappa^{2} }}$$

The values of Seebeck coefficients and the thermal conductivities of the TE legs were taken from results obtained from the annealed films measured above. The heat transfer coefficient for the heat transfer model considered is 12.16 W m^−2^ K^−1^ and it is based on Ref. [32]. The hot heat transfer surface area measures 1.44 mm^2^ and the cold heat transfer surface area considered is 0.46 mm^2^. Figure 6a and d show the predicted results of the TE devices found on Eqs. (4)–(11). In Fig. 6a, the open circuit voltage of the ultrathin TE devices increases upon an increase in the temperature difference. The difference between the simulated and experimental results for ST-BP reflects the cross-plane TE properties of TE modules, whereas the in-plane TE properties were used for analysis. The simulation results of ST-BA and ST-BT are in agreement with the measured values, indicating that the annealed Bi_2_Te_3_ and Bi_2_Te_3_/Au multilayers are isotropic. Although a more accurate simulation cannot be obtained due to the experimental limitations in the measurement of the cross-plane |*S*| of the thin film samples, these devices could be a way to evaluate the cross-plane Seebeck coefficient of thin film materials.

## Conclusions

In conclusion, 800-nm-thick n-type Bi_2_Te_3_, Bi_2_Te_3_/Au, Bi_2_Te_3_/Pt multilayers and p-type Sb_2_Te_3_ films were used to fabricate ultra-thin MEMS devices. The thermal stability of the evolution of the nanostructures and their corresponding thermoelectric properties before and after annealing were investigated. The Au compound in the Bi_2_Te_3_/Au multilayers agglomerates and limits the directional movement of the electrons, causing a significant drop in the conductivity and in the power factor. While the Bi_2_Te_3_/Pt multilayers still maintains a lamellar-like structure due to the excellent thermal stability of Pt, both the Seebeck coefficient and the conductivity increase. Therefore, a high-power factor of 46.5 μW cm^−1^ K^−2^ is obtained at 303 K by annealing the Bi_2_Te_3_/Pt multilayers to force the Pt layer to embed nano-inclusions into the Bi_2_Te_3_ matrix.

In addition, the thermoelectric conversion properties of the Bi_2_Te_3_ and Bi_2_Te_3_/(Au, Pt) multilayers were evaluated by measuring the properties of the MEMS devices. The output characteristics of the three devices are similar when the temperature is lower than 423 K. The no-load power of ST-BP reaches 20.9 nW, which corresponds to an enhancement larger than 39.5% when compared to ST-BT for a temperature higher than 423 K. This research facilitates the optimization of the microstructures and materials to fabricate thermoelectric modules and contributes to the development of novel applications of the low-dimensional thermoelectric materials in ultra-thin TE devices.

## Supplementary information

**Additional file 1:** Ultrathin MEMS thermoelectric generator with Bi2Te3/(Pt, Au) multilayers and Sb2Te3 legs.**Fig. S1.**Schematic outlining the basic steps of the fabrication processes for the ultrathin thermoelectric devices. (a) Positive photoresist coating on the substrate baking. (b) UV exposing (need align except for the first time). (c) Developing the exposure areas. (d) Depositing of bottom electrode. (e) Patterning using the lift-off technique. (f) UV exposing and depositing of TE modules. (g) Patterning using the lift-off technique. (h) UV exposing and depositing of another TE modules. (i) Patterning using the lift-off technique. (j) Supporting structure made by the vitrified photoresist. (k) UV exposing and depositing of top electrode. (l) Patterning using the lift-off technique. **Fig. S2.** Representative ΔV-ΔT curves of Sb2Te3, Bi2Te3 and Bi2Te3/(Au, Pt) multilayers; (a) Bi2Te3 before annealing, (b) Bi2Te3 after annealing, (c) Bi2Te3/Au multilayers before annealing, (d) Bi2Te3/Au multilayers after annealing, (e) Bi2Te3/Pt multilayers before annealing, (f) Bi2Te3/Pt multilayers after annealing, (g) Sb2Te3 before annealing, (h) S2Te3 after annealing. **Fig. S3.**Schematic diagram of the experimentally tested device.

## Data Availability

The datasets used and/or analyzed during the current study are available from the corresponding author on reasonable request.

## List of symbols

*A*_1_: contact area of the hot side (mm^2^)
*A*_2_: contact area of the cold side (mm^2^)
*B*: line broadening at half the maximum intensity (rad)
*D*: mean size of the ordered domains (nm)
*E*_A_: electron affinity (eV)
*E*_c_: the energy at the bottom of the conduction band (eV)
*E*_F_: Fermi level (eV)
*E*_g_: band gap (eV)
*E*_v_: the energy at the top of the valence band (eV)
*E*_vac_: vacuum level (eV)
*h*_1_: equivalent convection heat transfer coefficient of hot side (W m^−^^2^ K^−^^1^)
*h*_2_: equivalent convection heat transfer coefficient of cold side (W m^−^^2^ K^−^^1^)
*I*: open circuit output current of the TE device (A)
*k*_*B*_: Boltzmann constant
*K*: Scherrer constant
*n(E)*: carrier concentration (cm^−^^3^)
*q*: quantity of electric charge
*Q*_*C*_: rate of heat entering from the hot side of the TE device (W)
*Q*_*H*_: rate of heat leaving from the cold side of the TE device (W)
r: internal resistance value of TE device (Ω)
*S*: Seebeck coefficient (V K^−^^1^)
*T*: absolute temperature (K)
*T*_*C*_: cold side temperature (K)
*T*_*Cs*_: cold surface temperature of TE device (K)
*T*_*Hs*_: hot surface temperature of TE device (K)
*U*: open circuit output voltage of the TE device (V)
*v*: volume of the material (m^3^)

## Greek symbols

*α*_*v*_: volumetric thermal expansion coefficient
*ΔT*: temperature difference (K)
*Δv*: volume difference (cm^3^)
*ΔV*: voltage difference (V)
*σ(E)*: electrical conductivity (S cm^−^^1^)
*κ*: thermal conductivity (W m^−^^1^ K^−1^)
*μ(E)*: carrier mobility (cm^2^ V^−1^ s^−1^)
*θ*: Bragg angle (degree)
*Φ*: work function (eV)

## Abbreviations

DC: direct current
MEMS: microelectromechanical systems
*PF*: power factor
RF: radio frequency
SEM: scanning electron microscope
TDTR: time-domain thermoreflectance
TE: thermoelectric
XRD: X-ray diffraction
*ZT*: thermoelectric figure of merit

## References

[1] Cornett J, Chen B, Haidar S, Berney H, McGuinness P, Lane B, Gao Y, He Y, Sun N, Dunham M, Asheghi M, Goodson K, Yuan Y, Najafi K (2017). J. Electron. Mater..

[2] Yan J, Liao X, Yan D, Chen Y (2018). J. Microelectromech. S..

[3] Ran H, Schierning G, Nielsch K (2017). Adv. Mater. Technol..

[4] Xie J, Lee C, Feng H (2010). J. Microelectromech. S..

[5] Hadjistassou C, Kyriakides E, Georgiou J (2013). Energy. Convers Manage..

[6] Lim JR, Whitacre JF, Fleurial J-P, Huang C-K, Ryan MA, Myung NV (2005). Adv. Mater..

[7] Chen B, Kruse M, Xu B, Tutika R, Zheng W, Bartlett MD, Wu Y, Claussen JC (2019). Nanoscale.

[8] Bell LE (2008). Science.

[9] Kim SJ, We JH, Cho BJ (2014). Energy Environ. Sci..

[10] Minnich AJ, Dresselhaus MS, Ren ZF, Chen G (2009). Energy Environ. Sci..

[11] Zebarjadi M, Esfarjani K, Dresselhaus MS, Ren ZF, Chen G (2012). Energy Environ. Sci..

[12] Slack G (1995). CRC handbook of thermoelectrics.

[13] Hicks LD, Dresselhaus MS (1993). Phys. Rev. B.

[14] Hicks LD, Dresselhaus MS (1993). Phys. Rev. B.

[15] Venkatasubramanian R, Siivola E, Colpitts T, O'Quinn B (2001). Nature.

[16] Lee H, Anoop G, Lee H, Kim WS, Jo J (2019). RSC Advances.

[17] Dresselhaus MS, Chen G, Tang MY, Yang RG, Lee H, Wang DZ, Ren ZF, Fleurial J-P, Gogna P (2007). Adv. Mater..

[18] Sun T, Samani MK, Khosravian N, Ang KM, Yan Q, Tay BK, Hng HH (2014). Nano Energy.

[19] Sumithra S, Takas NJ, Misra DK, Nolting WM, Poudeu PFP, Stokes KL (2011). Adv. Energy Mater..

[20] Jeffrey SG, Lim JR, Huang C-K, Fleurial J-P (2003). Nat. Mater..

[21] Mu E, Wu Z, Wu Z, Chen X, Liu Y, Fu X, Hu Z (2019). Nano Energy.

[22] Mu E, Yang G, Fu X, Wang F, Hu Z (2018). J. Power Sources.

[23] Wu Z, Mu E, Wang Z, Chen X, Wu Z, Liu Y, Hu Z (2019). Cryst Growth Des.

[24] Chang G, Sun F, Duan J, Che Z, Wang X, Wang J, Kim MJ, Zhang H (2018). Acta Mater..

[25] Turcotte DL, Schubert G (2002). Geodynamics.

[26] Edwards AJ (1975). Anal. Chim. Acta.

[27] Faleev SV, Léonard F (2008). Phys. Rev. B.

[28] Zebarjadi M, Esfarjani K, Shakouri A, Bahk J-H, Bian Z, Zeng G, Bowers J, Lu H, Zide J, Gossard A (2009). Appl. Phys. Lett..

[29] Lide DR (2008). CRC Handbook of Chemistry and Physics.

[30] Shen Z-G, Wu S-Y, Xiao L, Yin G (2016). Energy.

[31] Chen L, Gong J, Sun F, Wu C (2002). Int. J. Therm. Sci..

[32] Siddique ARM, Rabari R, Mahmud S, Heyst BV (2016). Energy.

