# Supplementary material for: Ultrathin MEMS thermoelectric generator with Bi2Te3/(Pt, Au) multilayers and Sb2Te3 legs
Source: Nano Converg. 2020 Mar 3;7:8. doi: 10.1186/s40580-020-0218-x (PMC7052102; doi:10.1186/s40580-020-0218-x)
Supplement: Supplementary file 1 — Additional file 1: Ultrathin MEMS thermoelectric generator with Bi2Te3/(Pt, Au) multilayers and Sb2Te3 legs.Fig. S1.Schematic outlining the basic steps of the fabrication processes for the ultrathin thermoelectric devices. (a) Positive photoresist coating on the substrate baking. (b) UV exposing (need align except for the first time). (c) Developing the exposure areas. (d) Depositing of bottom electrode. (e) Patterning using the lift-off technique. (f) UV exposing and depositing of TE modules. (g) Patterning using the lift-off technique. (h) UV exposing and depositing of another TE modules. (i) Patterning using the lift-off technique. (j) Supporting structure made by the vitrified photoresist. (k) UV exposing and depositing of top electrode. (l) Patterning using the lift-off technique. Fig. S2. Representative ΔV-ΔT curves of Sb2Te3, Bi2Te3 and Bi2Te3/(Au, Pt) multilayers; (a) Bi2Te3 before annealing, (b) Bi2Te3 after annealing, (c) Bi2Te3/Au multilayers before annealing, (d) Bi2Te3/Au multilayers after annealing, (e) Bi2Te3/Pt multilayers before annealing, (f) Bi2Te3/Pt multilayers after annealing, (g) Sb2Te3 before annealing, (h) S2Te3 after annealing. Fig. S3.Schematic diagram of the experimentally tested device. [file 40580_2020_218_MOESM1_ESM.doc]

**supplementary materials**

# Ultrathin MEMS thermoelectric generator with Bi_2_Te_3_/(Pt, Au) multilayers and Sb_2_Te_3_ legs

**
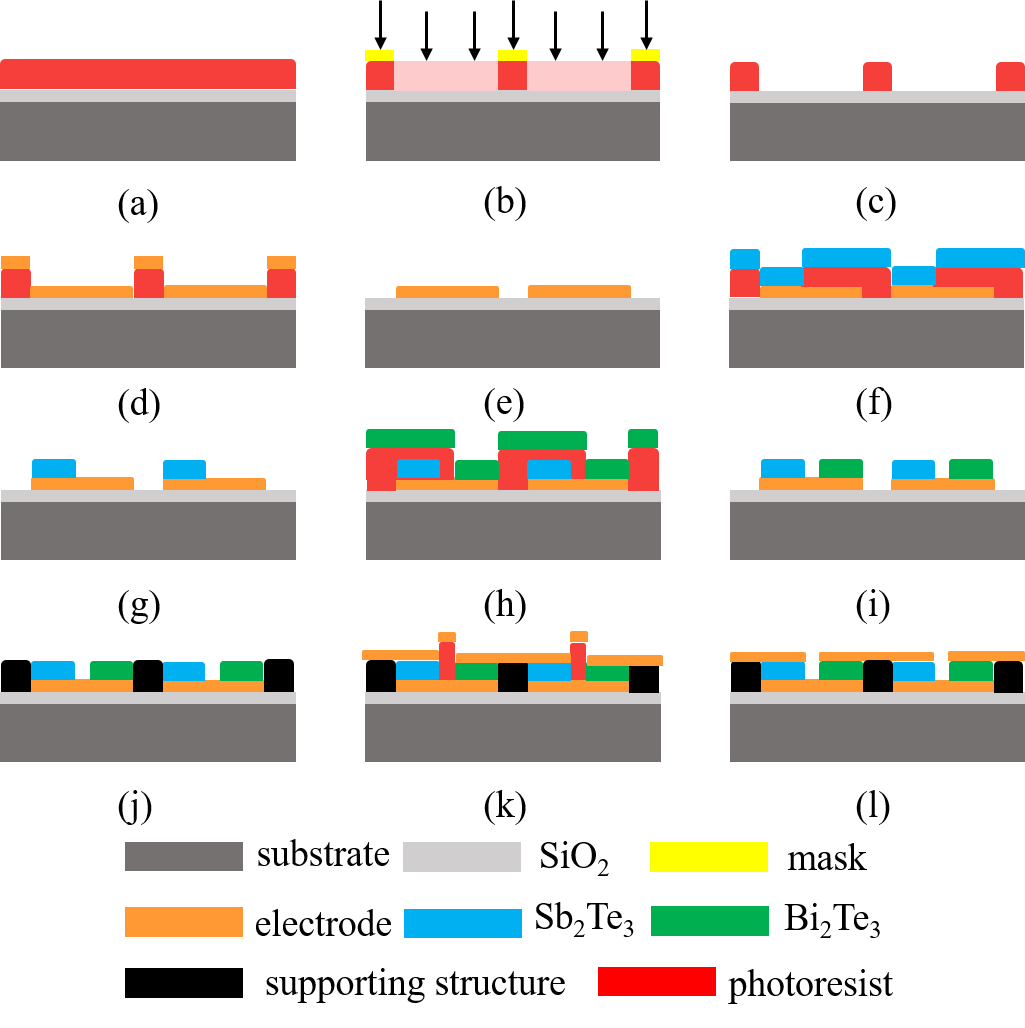
**

Fig. S1 Schematic outlining the basic steps of the fabrication processes for the ultrathin thermoelectric devices. (a) Positive photoresist coating on the substrate baking. (b) UV exposing (need align except for the first time). (c) Developing the exposure areas. (d) Depositing of bottom electrode. (e) Patterning using the lift-off technique. (f) UV exposing and depositing of TE modules. (g) Patterning using the lift-off technique. (h) UV exposing and depositing of another TE modules. (i) Patterning using the lift-off technique. (j) Supporting structure made by the vitrified photoresist. (k) UV exposing and depositing of top electrode. (l) Patterning using the lift-off technique.


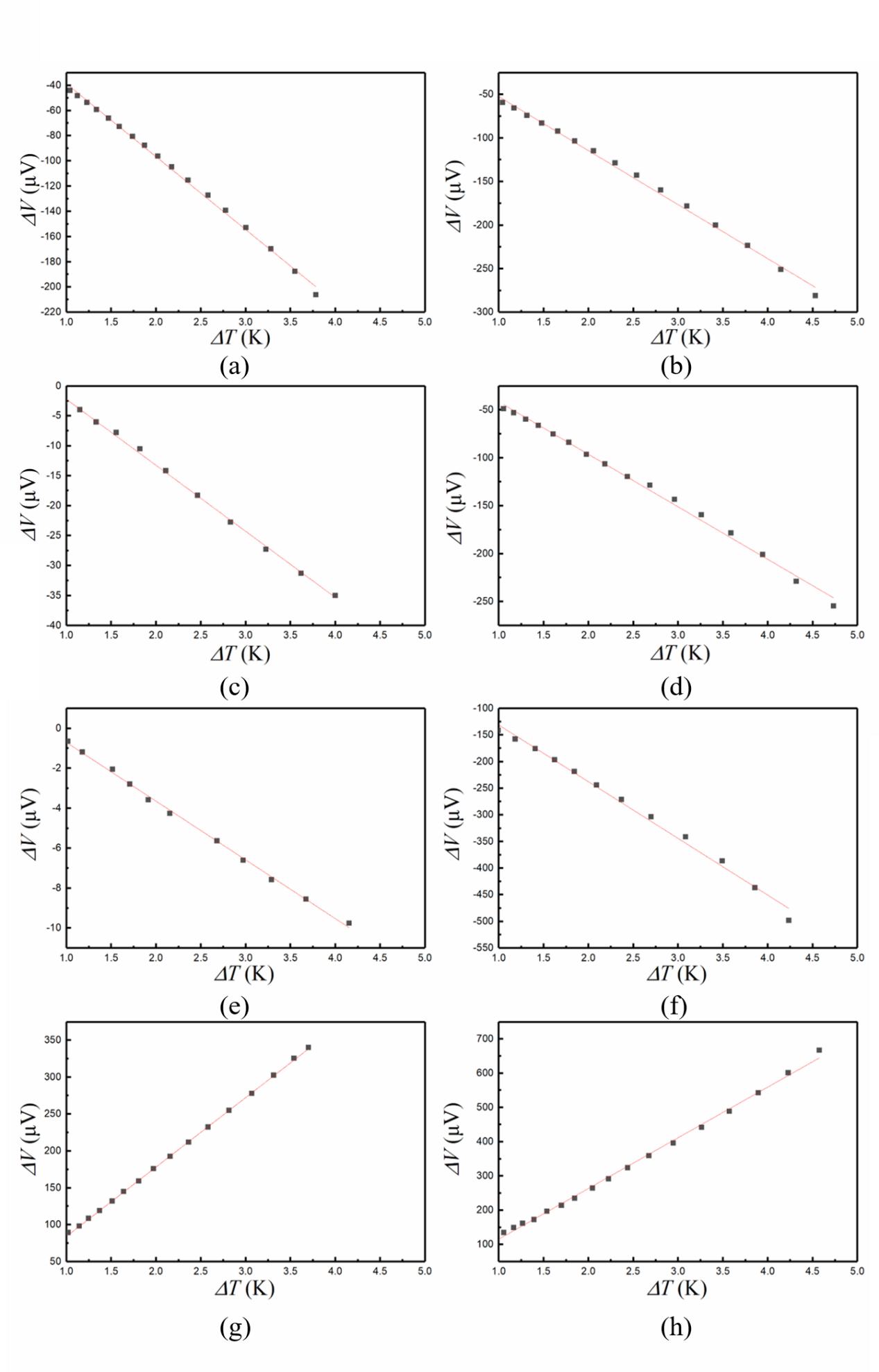


Fig. S2 Representative ΔV-ΔT curves of Sb_2_Te3, Bi_2_Te_3_ and Bi_2_Te_3_/(Au, Pt) multilayers; (a) Bi_2_Te_3_ before annealing, (b) Bi_2_Te_3_ after annealing, (c) Bi_2_Te_3_/Au multilayers before annealing, (d) Bi_2_Te_3_/Au multilayers after annealing, (e) Bi_2_Te_3_/Pt multilayers before annealing, (f) Bi_2_Te_3_/Pt multilayers after annealing, (g) Sb_2_Te_3_ before annealing, (h) S_2_Te_3_ after annealing


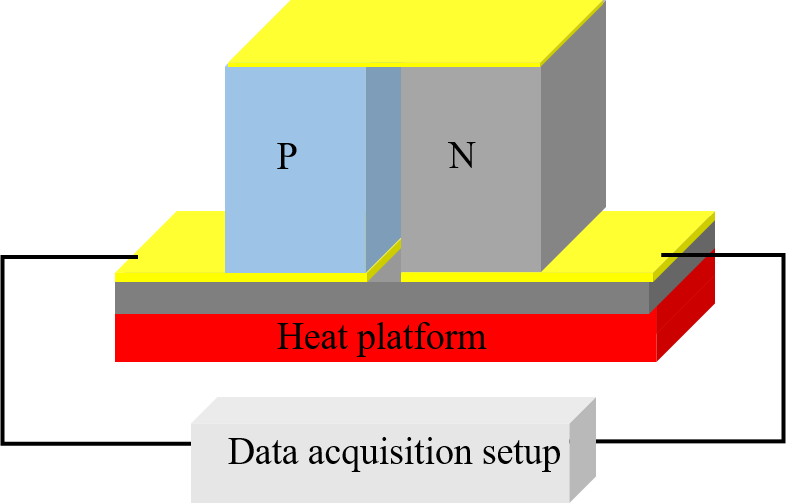


Fig.S3 Schematic diagram of the experimentally tested device.
